# Petite Integration Factor 1 (PIF1) helicase deficiency increases weight gain in Western diet-fed female mice without increased inflammatory markers or decreased glucose clearance

**DOI:** 10.1371/journal.pone.0203101

**Published:** 2019-05-28

**Authors:** Frances R. Belmonte, Nikolaos Dedousis, Ian Sipula, Nikita A. Desai, Aatur D. Singhi, Yanxia Chu, Yingze Zhang, Sylvie Bannwarth, Véronique Paquis-Flucklinger, Lea Harrington, Sruti Shiva, Michael J. Jurczak, Robert M. O’Doherty, Brett A. Kaufman

**Affiliations:** 1 University of Pittsburgh School of Medicine, Division of Cardiology, Center for Metabolism and Mitochondrial Medicine, and Vascular Medicine Institute, Pittsburgh, PA, United States of America; 2 Department of Medicine, Division of Endocrinology and Metabolism, University of Pittsburgh, Biomedical Science Tower, Pittsburgh, PA, United States of America; 3 Department of Pathology and Pittsburgh Liver Research Center, University of Pittsburgh, Scaife Hall, Pittsburgh, PA, United States of America; 4 Department of Medicine, Division of Pulmonary, Allergy and Critical Care Medicine, UPMC Montefiore Hospital, Pittsburgh, PA, United States of America; 5 Université Côte d'Azur, CHU de Nice, Inserm, CNRS, IRCAN, France; 6 Université de Montréal, Institut de Recherche en Immunologie et en Cancérologie, Montréal, Québec, Canada; University of Melbourne, AUSTRALIA

## Abstract

Petite Integration Factor 1 (PIF1) is a multifunctional helicase present in nuclei and mitochondria. PIF1 knock out (KO) mice exhibit accelerated weight gain and decreased wheel running on a normal chow diet. In the current study, we investigated whether *Pif1* ablation alters whole body metabolism in response to weight gain. PIF1 KO and wild type (WT) C57BL/6J mice were fed a Western diet (WD) rich in fat and carbohydrates before evaluation of their metabolic phenotype. Compared with weight gain-resistant WT female mice, WD-fed PIF1 KO females, but not males, showed accelerated adipose deposition, decreased locomotor activity, and reduced whole-body energy expenditure without increased dietary intake. Surprisingly, PIF1 KO females did not show obesity-induced alterations in fasting blood glucose and glucose clearance. WD-fed PIF1 KO females developed mild hepatic steatosis and associated changes in liver gene expression that were absent in weight-matched, WD-fed female controls, linking hepatic steatosis to *Pif1* ablation rather than increased body weight. WD-fed PIF1 KO females also showed decreased expression of inflammation-associated genes in adipose tissue. Collectively, these data separated weight gain from inflammation and impaired glucose homeostasis. They also support a role for *Pif1* in weight gain resistance and liver metabolic dysregulation during nutrient stress.

## Introduction

The increasing prevalence of obesity is a global public health problem, which has been particularly striking among children and adolescents in both developed and developing countries [1]. Clinical complications of obesity include metabolic syndrome, which can progress to type 2 diabetes mellitus (T2DM) and cardiovascular disease. Current understanding of the genes involved in obesity is limited, despite its high heritability [2]. Approximately 90% of patients with T2DM are overweight or obese. However, while being overweight greatly increases the likelihood of developing T2DM [3], only a subset of obese individuals acquires T2DM. Factors such as tissue inflammation that drive obesity or that determine the diabetic phenotype in obese individuals are poorly understood.

Mouse models have been used to investigate genetic factors that alter the incidence and severity of obesity and diabetes in states of over nutrition, such as from a Western diet (WD) characterized by a high fat and carbohydrate content. We previously observed that male and female Petite Integration Frequency 1 helicase knockout (PIF1 KO) mice had increased body weight on regular chow [4]. This suggested that *Pif1* could play a role in the pathogenesis of obesity, although it remains unclear how *Pif1* contributes to body weight maintenance. PIF1 is a conserved ATP-dependent RNA/DNA helicase that is ubiquitously expressed at low levels in tight coordination with the cell cycle [5] and localizes to both the nucleus and mitochondria [6]. In yeast, Pif1p inhibits telomerase [7–10], resolves short flaps in replication forks and unwinds secondary genomic structures that interfere with nuclear replication elongation [11, 12]. Mammalian PIF1 also interacts with telomerase [13]. However, in that study the PIF1 KO mice exhibited normal telomere length after four generations of homozygosity, suggesting a different mechanism for this protein in metabolism.

The physiological basis for *Pif1* regulation of body weight has not been addressed. The primary objective of this study was to determine whether WD-fed PIF1 KO mice develop metabolic alterations under diet-induced weight gain. Toward that end, we measured major determinants of energy balance including locomotor activity, feeding, and energy expenditure by indirect calorimetry. Body composition, glucose tolerance and inflammation were also assessed. We found that PIF1 KO females, but not males, gained more weight on a WD, which was attributed specifically to increased fat mass. Surprisingly, PIF1 KO female mice were protected against the glucose intolerance and inflammation that are typically associated with obesity. The physiological basis for PIF1 KO female fat deposition was likely due to changes in physical activity. Our findings highlight an important role for a helicase such as PIF1 in regulating metabolism.

## Materials and methods

### Animals

Wild type C57BL/6J mice were from the Jackson Laboratory (Bar Harbor, ME). PIF1 KO mice were originally developed in the C57BL/6 background [4, 13], but the *Pif1* deletion was maintained in the C57BL/6J background exclusively for more than 10 generations. Animals were fed normal or WD chow *ad libitum* and housed in a 12 h light to dark cycling room. This study was performed in strict accordance with the recommendations in the “Guide for the Care and Use of Laboratory Animals” (2018) of the National Institutes of Health (Bethesda, MD) and was approved by the Institutional Animal Care and Use Committee of the University of Pittsburgh (Protocol Numbers: 15035458 and 18032212).

**Fig 1:**
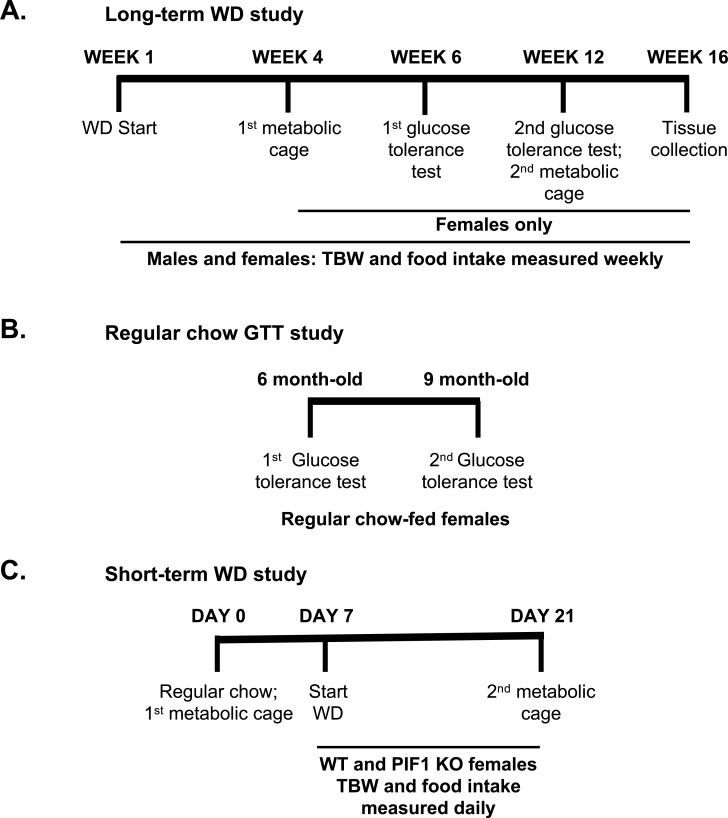
Experimental timelines for each cohort. A) Long-term Western diet (WD) study. Starting at 2 months of age, males and females undergo WD feeding with weekly measurements of total body weight (TBW). Females also had food intake, metabolic cage experiments, and glucose tolerance tests at the indicated weeks. B) A second cohort of 2-month-old, regular chow-fed WT and PIF1 KO females underwent glucose tolerance tests at 6 and 9 months of age. C) A separate cohort of 9-week-old WT and PIF1 KO females were fed regular chow and were used in the first metabolic cage experiments on Day 0 (TBW and food intake measured daily through Day 21), followed by the start of WD feeding on Day 7 through Day 21. The mice were analyzed in a second metabolic cage assessment and subsequently euthanized.

Three different animal cohorts were used for experiments as shown in the timelines (Fig 1A–1C). The experimental cohort for long-term WD studies was comprised of eight- to nine-week-old WT and PIF1 KO males and females exclusively fed a WD (41% fat, 27% sucrose, 19% protein, per calories; no. 96001; Harlan Teklad, Madison, WI) for 16 weeks (8–12 per group). Four mice of the same sex, genotype and age were group housed together. Total body weight for all mice was measured weekly for 16 weeks. Separately, a second cohort consisted of 2-month-old WT and PIF1 KO female mice that were fed a regular chow diet (14% fat, 60% carbohydrates, 26% protein; no. 5P76; LabDiet, St. Louis, MO.) beginning at the time of weaning at 1 month of age through 9 months (10 per group). A third experimental cohort for short-term WD studies consisted of nine-week-old, age- and weight-matched WT and PIF1 KO female mice (eight per group) that were singly housed to allow measurement of individual food intake and weight.

For the short-term WD feeding study, baseline metabolic measurements (as described in the following section) were obtained from female mice fed regular chow before transitioning to the WD. Metabolic measurements were repeated 14 days after WD initiation. Total body weight and WD intake were measured daily, Monday through Friday. Food intake was calculated per mouse, per total body weight, or per lean mass as indicated.

### Metabolism and body composition

Metabolic profiles of WT and PIF1 KO mice in both long- and short-term studies (at least eight per group for each study) were compared using the Comprehensive Laboratory Animal Monitoring System (CLAMS, Columbus Instruments, Columbus, OH). Mice were acclimated for 24 h then energy expenditure, foraging activity, oxygen consumption and carbon dioxide production were measured for a second 24 h period. Locomotor activity was measured by averaging the total beam break counts along the x-axis from each animal per genotype. Animals underwent body composition analyses using ^1^H magnetic resonance spectroscopy (EchoMRI-100H, Houston, TX).

### Serum cytokine, diabetes, obesity, and cholesterol concentrations

WT and PIF1 KO females were fasted for 6 h prior to euthanasia. Blood was collected by cardiac puncture and centrifuged at 1000 x g for 10 min. Serum concentrations of cytokines (IL-1α, IL-1β, IL-12, eotaxin, G-CSF, IFN-γ, MCP-1, MIP-1β, TNF-α, IL-12p70), diabetes and obesity markers (ghrelin, gastric inhibitory polypeptide, glucagon-like peptide-1, glucagon, insulin, leptin, plasminogen activator inhibitor-1, resistin) were quantified with the Bio-Plex Pro Mouse Cytokine 23-plex and Diabetes 8-plex immunoassays, respectively (Bio-Rad, Hercules, CA). Total cholesterol, high-density lipoprotein and low-density lipoprotein/ very low-density lipoprotein serum concentrations were measured using colorimetric methods described in the manufacturer’s protocol (BioAssay Systems, Hayward, CA).

### Intraperitoneal glucose tolerance test

Six- and nine-month-old, regular chow-fed female WT and PIF1 KO mice (10 per group) and a separate cohort of WD-fed, 3.5- and five-month-old female WT and PIF1 KO mice (8–12 per group) were fasted for 6 h before measuring baseline glucose concentrations in whole blood sampled from a tail vein. D-glucose diluted in saline was administered at 1 g/kg body weight by intraperitoneal injection and blood glucose concentrations measured every 15 min for 2 h using Contour blood glucose test strips (Ascensia Diabetes Care US Inc, Parsippany, NJ) inserted into a glucometer.

### Liver pathology

Livers from female WT and PIF1 KO mice (9–10 per group) were fixed in 10% formalin for 24 h, transferred in ethanol and paraffin embedded before sectioning and staining. Liver sectioning and haematoxylin and eosin staining were performed at the Transplant Surgery Experimental Pathology Laboratory, Research Histology Services Core at the University of Pittsburgh. Liver histopathology was scored using a standardized scale (https://tpis.upmc.com/changebody.cfm?url=/tpis/schema/NAFLD2006.jsp) [3] performed by a surgical pathologist blinded to the experimental cohort.

### Mitochondrial DNA (mtDNA) copy content and mitochondrial complex I activity

Total DNA from liver was prepared as described in [14] and was used for multiplex assessment of mtDNA copy content via quantitative PCR (qPCR) assay. TaqMan Universal Master Mix (Applied Biosystems, Waltham, MA), DNA, and 5 μM of ND1 and B2M primer/probes were combined in 15 μL reactions; qPCR assays were performed on a StepOnePlus thermocycler (Applied Biosystems) using the following standard thermal parameters: 50°C for 2 min, 95°C for 10 min and 40 cycles of 95°C for 15 sec and 60°C for 1 min. MtDNA copy content was calculated using the ΔΔC_q_ method [15].

Mitochondrial complex I activity was measured in liver homogenates as described in [16]. Briefly, powered liver tissue was homogenized in cold sodium chloride-Tris-EDTA (STE) buffer; homogenates were transferred to 1.5 mL centrifuge tubes. The freeze-thaw method was applied to the liver homogenates to lyse the cells; 5 freeze/thaw cycles were performed using liquid nitrogen followed by thawing at 37° Celsius. The homogenates were subsequently centrifuged for 10 minutes at 20,000 x g (4°C). Supernatants were collected and protein content was measured using the bicinchoninic acid assay. For the complex I activity assay, supernatants were diluted at a concentration of 5 μg/μL in STE buffer. Complex I activity was measured colorimetric assay (decylubiquinone served as an electron acceptor that is converted to decylubiquinol through Complex I catalytic activity).

### Quantitative reverse transcription PCR

Total RNA was isolated from powdered gonadal white adipose and liver tissues using TRIzol extraction (Invitrogen, Carlsbad, CA), and purified on a RNeasy column (Qiagen, Germantown, MD) [17]. Potential DNA contamination was removed by a DNA-free assay (ThermoFisher). Random hexamer primed reverse transcription was performed using the High Capacity cDNA synthesis kit as per the manufacturer’s protocol (ThermoFisher). Quantitative real time PCR was performed on a QuantStudio 5 Real-Time PCR System (ThermoFisher) using PowerUp SYBR Green (ThermoFisher) or TaqMan Fast Advanced Mastermix (ThermoFisher) when using Integrated DNA technologies (IDT; Coralville, IA) Assay Mm.PT.58.31244167. Reaction setup was performed using a Tecan Evo150 automated platform (Tecan, Morrisville, NC). Digital PCR of Mm.PT.58.31244167 was then performed on a pooled standard using the QuantStudio 3D Digital PCR Master Mix v2 on a QuantStudio 3D Digital PCR System (Thermofisher). Absolute values of PIF1 transcript copies per microgram of RNA was then calculated using the data from the digital PCR and applied to the quantitative PCR results. Primers used in this study are presented in S1 Table.

### Statistical analysis

Statistical analyses were performed using GraphPad Prism 7 software (GraphPad, La Jolla, CA). Student’s two-tailed, unpaired t-test was used to determine the significance of differences between two groups. Additionally, the student’s two-tailed, unpaired t-test was applied to assess significant differences in the rate of weight gain between two groups. Two-way repeated measures ANOVA with indicated post-hoc multiple comparisons test were used to compare values obtained from weight gain, metabolic cage data, and to assess the factors of genotype and light/dark cycle on locomotor activity and whole-body energy expenditure. Area under the concentration-time curve (AUC) analyses were performed to determine the total areas under the glucose tolerance test curves. P-values of <0.05 denoted significant differences. Standard error of the mean is shown for all experiments.

## Results

### Female PIF1 KO mice gained weight and increased fat mass faster than WT mice when fed a WD

**Fig 2:**
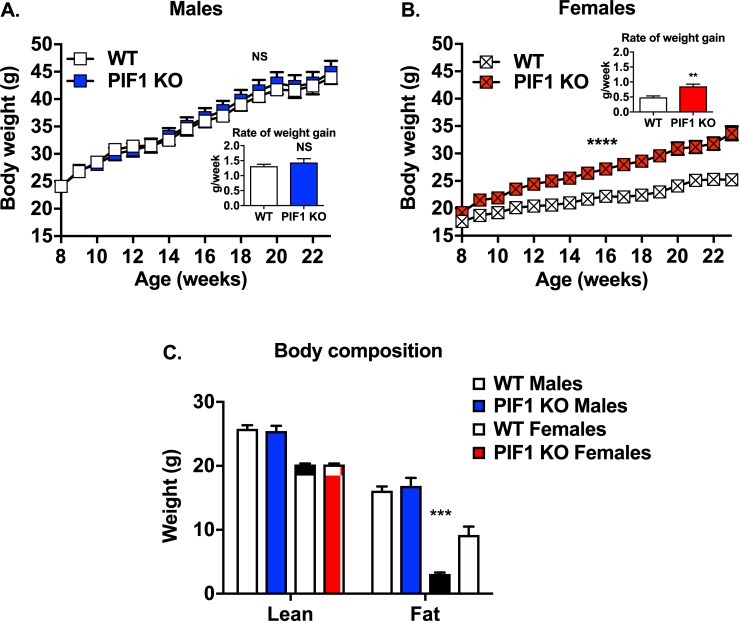
PIF1 knockout (KO) females, but not males, showed accelerated fat deposition compared with wild type (WT) mice during Western diet (WD) feeding. A) Average weekly body weight during 16 weeks of WD feeding in WT and PIF1 KO male (n = 12 and 11, respectively), or B) WT and PIF1 KO female mice (n = 8 and 12, respectively). Significance is reported for two-way ANOVA with repeated measures. Inset: Rate of body weight gain was calculated for each animal as a single value and used to determine p-values *via* Student’s t-test. C) Body composition as assessed through lean and fat mass measured in male WT (n = 11) and PIF1 KO (n = 11), male and female, 5-month-old mice after 12 weeks of WD feeding. Shown are mean ± SEM; p-values are calculated by Two-way ANOVA with Tukey’s posthoc test for multiple comparisons, shown are values for same gender WT vs PIF1 KO: **p<0.01; ***p<0.001; g = grams.

To further define the role of *Pif1* in regulating body weight, we fed male and female WT and PIF1 KO mice a WD for 16 weeks (characterized as long-term WD feeding) to induce weight gain (Fig 1A cohort). PIF1 KO and WT males gained weight rapidly at similar rates (Fig 2A), while body composition was similar in the two groups (Fig 2C). In contrast, PIF1 KO females gained significantly more weight and at a faster rate than WT females (Fig 2B). Body composition analyses demonstrated that these differences were due exclusively to increases in fat mass in WD-fed PIF1 KO females, with lean mass being the same in the two groups (Fig 2C). As the weight of WD-fed PIF1 KO females diverged from WT females, we focused exclusively on the female cohort in subsequent experiments.

### PIF1 KO female mice fed a WD for 16 weeks (long-term) showed decreased locomotor activity compared with WT female mice

**Fig 3:**
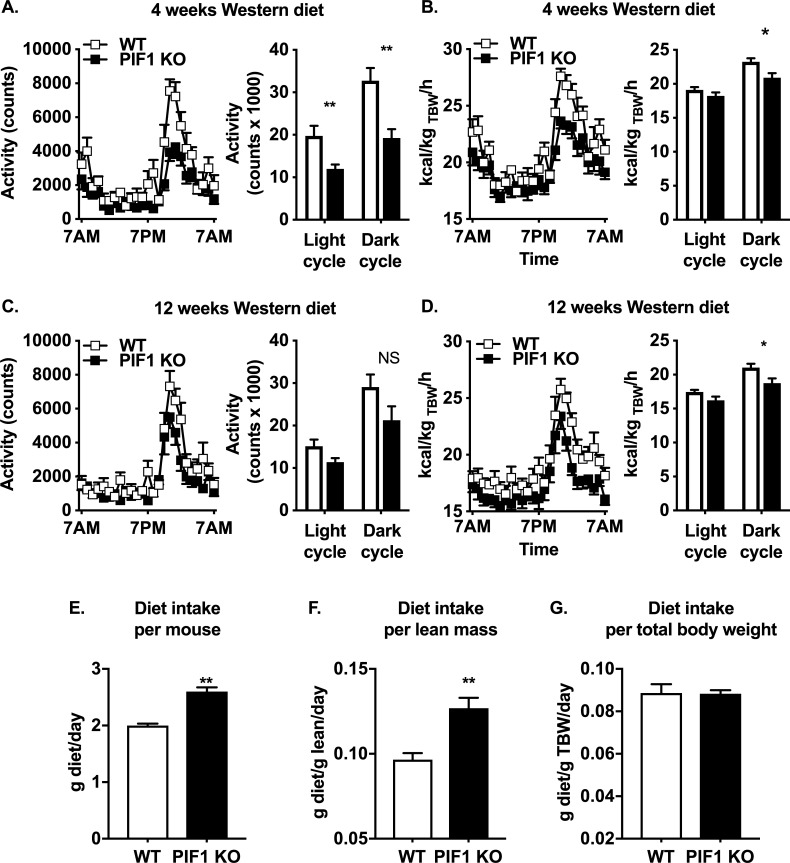
Long-term, Western diet (WD)-fed PIF1 KO female mice exhibited decreased locomotor activity and whole-body energy expenditure. A) Locomotor activity and B) whole-body energy expenditure measurements in 3-month-old, PIF1 KO female mice after 4 weeks of WD feeding. C) Locomotor activity and D) whole-body energy expenditure measured in 5-month-old, WD-fed PIF1 KO female mice after 12 weeks of feeding. E) WD food intake per mouse determined as the average of weeks 13–16 WD intake for each mouse. F) WD food intake normalized to lean mass. G) WD food intake normalized to total body weight across. All data are mean ± SEM; n = 8 mice per group; Student’s T-test for pvalues: *p<0.05, **p<0.01; kcal/kg TBW/h = kilocalories per kilogram total body weight per hour; g = grams.

To understand the physiological basis of body weight differences between WD-fed PIF1 KO and WT female mice, we measured the major determinants of energy balance (Fig 1A cohort). After four weeks of WD feeding, three-month-old PIF1 KO females showed reduced locomotor activity during both light and dark cycles and decreased whole-body energy expenditure across 24 h (Fig 3A and 3B). Following 12 weeks of WD feeding, 5-month-old PIF1 KO females trended toward decreased locomotor activity (Fig 3C), but the WT animal activity began to decrease with weight gain preventing statistical significance between the two groups. Whole-body energy expenditure remained lower in 5-month-old PIF1 KO females during the dark cycle (Fig 3D). Substrate utilization, determined by the respiratory exchange ratio, was not different at any time point.

To assess whether decreased locomotion preceded weight gain, a separate cohort of age- and weight-matched WT and PIF1 KO female mice were fed a regular chow diet and then placed on a 14-day (short-term) WD diet. Activity was measured in both dietary conditions. On regular chow (Fig 1C cohort, Day 0), locomotor activity and whole-body energy expenditure were not different between groups (S1A and S1B Fig). After 14 days of WD, locomotor activity trended downward during the dark cycle in PIF1 KO females compared with WT females (S1C Fig), while energy expenditure did not differ between groups (S1D Fig). Thus, the decreased locomotor activity observed in the long-term WD-fed PIF1 KO females preceded the weight gain.

To determine whether WD was a potential driver for the weight gain phenotype in WD-fed PIF1 KO females, we assessed food consumption in the long- and short-term WD studies (Fig 1A and 1C, respectively). Total WD intake per mouse (Fig 3E) or normalized to lean mass (Fig 3F) were higher overall in WD-fed PIF1 KO versus WT females, but not when normalized to lean body mass (Fig 3G). Differences in body weight can confound interpretation of feeding data [18]. Therefore, we measured WD intake under body-weight matched conditions using short-term WD-fed animals and found no differences in feeding (S1E Fig). Thus, increased body weight in WD-fed PIF1 KO female mice was unlikely to be due to differences in food intake.

### Glucose tolerance is unchanged in WD-fed PIF1 KO females despite increased adiposity

**Fig 4:**
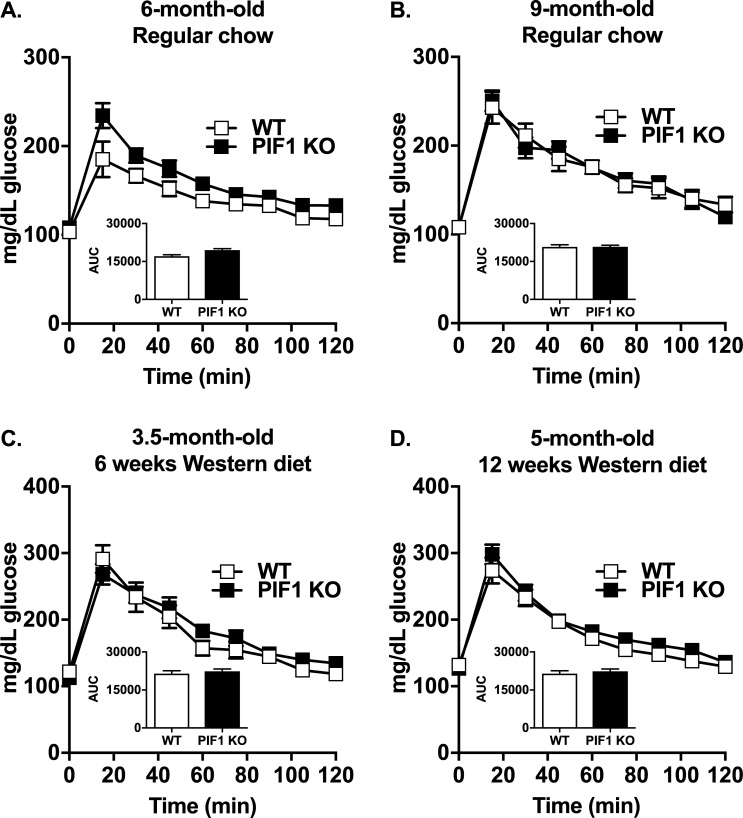
Western diet (WD) feeding did not affect blood glucose clearance in regular chow- and WD-fed PIF1 KO mice. A) Glucose tolerance tests were performed on six-month-old WT and PIF1 KO mice after 20 weeks of regular chow feeding, and on B) the same mice at nine months of age after 32 weeks of regular chow feeding. C) Glucose tolerance tests conducted on 3.5-month-old WT and PIF1 KO mice after 6 weeks of WD feeding, and D) 5-month-old WT and PIF1 KO mice after 12 weeks of WD feeding. All data are mean ± SEM; n = 8–12 mice per group; mg/dl = milligrams per deciliter; AUC = area under the curve. No data are significant by Student’s T-test.

As body weight strongly influences fasting blood glucose concentrations and glucose tolerance, we performed intraperitoneal glucose tolerance tests on regular chow- and WD-fed WT and PIF1 KO female mice in separate experiments (cohorts from Fig 1A and 1B, respectively). Body weight-matched six-month-old (Fig 4A) and nine-month-old (Fig 4B) regular chow-fed females exhibited normal fasting blood glucose concentrations and glucose tolerance. Interestingly, despite the increased body weight and adiposity in long-term WD-fed PIF1 KO female mice (Fig 2), fasting blood glucose and glucose tolerance were not impaired relative to WT females after 6 and 12 weeks of WD-feeding (Fig 4C and 4D).

The apparent protection against insulin resistance in PIF1 KO female mice prompted us to examine specific serum factors that are commonly dysregulated during diabetes and obesity (Table 1). Consistent with normal glucose tolerance, fasting insulin concentrations were similar between genotypes after long-term WD feeding (cohort from Fig 1A, 16 weeks WD). As expected with the increased adiposity in the PIF1 KO mice, leptin concentrations were significantly elevated in serum. Other factors associated with diabetes and obesity including ghrelin, gastric inhibitory polypeptide (GIP), glucagon-like peptide-1 (GLP-1), plasminogen activator inhibitor-1 (PAI-1), resistin, cholesterol, high-density lipoprotein (HDL) and low-density lipoprotein/ very low-density lipoprotein (LDL/VLDL) were largely unchanged. Glucagon, a metabolic hormone that is secreted during fasting and is a positive regulator of gluconeogenesis, was upregulated. This likely occurs to maintain normoglycemia in obese animals in the fasted state [19].

**Table 1:** Diabetes, obesity, and cholesterol concentrations in serum from Western diet-fed, WT and PIF1 KO female mice.

Marker	WT	PIF1 KO
Ghrelin (ng/mL)	3.67 ± 0.56	2.57 ± 0.31
GIP (ng/mL)	2.43 ± 0.82	2.57 ± 0.31
GLP-1 (ng/mL)	0.033 ± 0.021	0.006 ± 0.004
Glucagon (ng/mL)	0.074 ± 0.015	0.306 ± 0.078*
Insulin (ng/mL)	3.32 ± 1.78	1.62 ± 0.40
Leptin (ng/mL)	4.80 ± 1.84	23.32 ± 4.43**
PAI-1 (ng/mL)	1.17 ± 0.18	1.46 ± 0.06
Resistin (ng/mL)	190.3 ± 24.01	200.02 ± 21.71
Total cholesterol (mg/dL)	305.33 ± 56.24	394.86 ± 80.72
HDL (mg/dL)	297.30 ± 62.85	223.16 ± 50.36
LDL/VLDL (mg/dL)	207.15 ± 60.64	231.69 ± 67.44

All values are mean ± SEM; n = 3–12 per group; pvalues from Student’s t-test:

*p<0.05

**p<0.01.

### WD-fed PIF1 KO female mice exhibited mild hepatic steatosis with modest alterations in markers of gluconeogenesis and mitochondrial biogenesis

**Fig 5:**
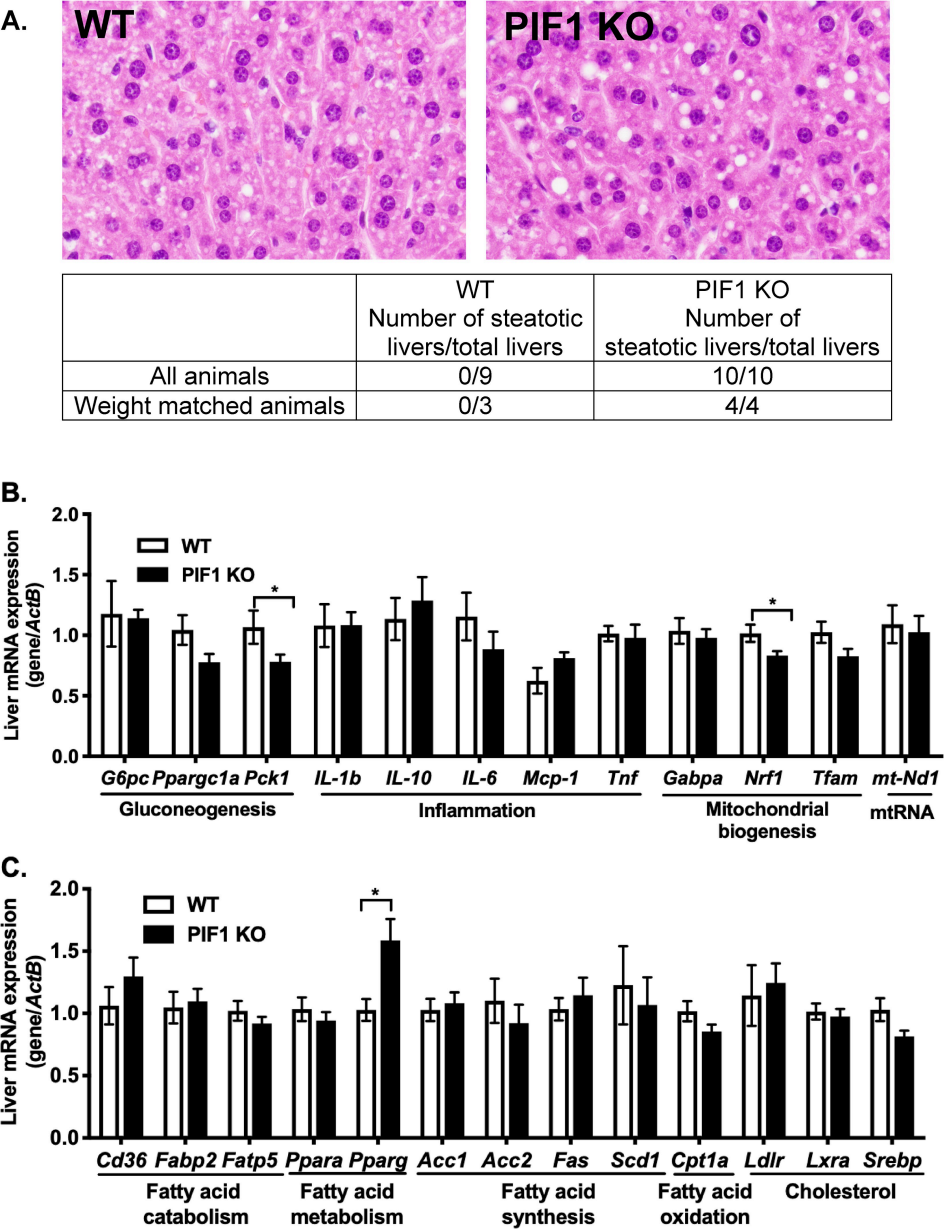
Livers from long-term, Western diet (WD)-fed PIF1 KO female mice showed minor macro hepatic steatosis and altered transcripts of steatosis and gluconeogenesis markers. A) Representative images of haematoxylin and eosin stained livers from WD-fed WT and PIF1 KO females (n = 9 and 10 mice per group, respectively). The table below shows the total number of livers from all animals and weight-matched, WT and PIF1 KO livers that were blindly scored for the presence of macro hepatic steatosis. B) Liver cDNA from WD-fed, WT and PIF1 KO mice was used to measure the expression of genes involved in gluconeogenesis, inflammation, mitochondrial biogenesis, mitochondrial RNA, and C) fatty acid catabolism, fatty acid metabolism, fatty acid synthesis, fatty acid oxidation and cholesterol. mRNA expression relative to beta actin (*ActB*) reference gene in liver tissue. All data are mean ± SEM; n = 8–12 mice per group; pvalues determined by Student’s T-test, *p<0.05.

During weight gain, hepatic fat accumulation can lead to alterations in fatty acid metabolism and cholesterol handling. To investigate whether PIF1 KO female mice were particularly vulnerable to hepatic steatosis, haematoxylin and eosin stained liver sections from the long-term WD cohort (cohort from Fig 1A) were evaluated in a blinded fashion by a liver pathologist. PIF1 KO female mice uniformly exhibited mild hepatic steatosis compared to WT mice (Fig 5A). To determine whether differences in steatosis were due to obesity, we compared a weight-matched subset of animals (3 WT and 4 PIF1 KO) and found steatosis only in the PIF1 KO animals. These data indicate that the hepatic steatosis observed in WD-fed PIF1 KO mice was independent of weight gain.

To examine differences in the transcriptional response between long-term WD-fed WT and PIF1 KO livers (cohort from Fig 1A), we analyzed the expression of a set of genes with established roles (gluconeogenesis, inflammation, mitochondrial biogenesis and lipid metabolism) in liver fat accumulation (Fig 5B and 5C). Transcripts for phosphoenolpyruvate carboxylkinase 1 (*Pck1*), a rate-limiting enzyme for gluconeogenesis, and nuclear respiratory factor 1 (*Nrf1*), a transcription factor involved in regulating mitochondrial biogenesis, were decreased in livers from WD-fed PIF1 KO mice (Fig 5B). Expression of genes known to regulate gluconeogenesis *(G6pc*, *Ppargc1a)*, inflammation *(Il-1b*, *Il-10*, *Il-6*, *Mcp-1*, *Tnf)*, mitochondrial biogenesis *(Gabpa*, *Tfam)* or mitochondrial DNA encoded genes (*mt-Nd1*) were similar between groups (Fig 5B). Additionally, we did not observe any differences in mitochondrial DNA (mtDNA) copy number or mitochondrial complex I activity between WD-fed WT and PIF1 KO female livers (S2B Fig). PIF1 KO livers showed increased expression of peroxisome proliferator-activated receptor gamma (*Pparg*) (Fig 5C), consistent with the steatotic phenotype [20–25]. Other genes that regulate fatty acid and cholesterol metabolism (*Cd36*, *Fabp2*, *Fatp5*, *Ppara*, *Acc1*, *Acc2*, *Fas*, *Scd1*, *Cpt1a*, *Ldlr*, *Lxra*, *and Srebp*) were unchanged in WT and PIF1 KO livers. No difference in PIF1 transcript numbers in liver from regular chow and WD-fed female WT mice we detected to indicate dietary regulation of the gene (S3 Fig). Overall, these results showed that WD-fed PIF1 KO female mice developed mild hepatic steatosis with modest gene expression changes in relevant metabolic pathways.

### Adipose tissue from WD-fed PIF1 KO females has decreased mRNA expression of inflammatory markers without changes in markers of adiposity, fatty acid metabolism and mitochondrial biogenesis

**Fig 6:**
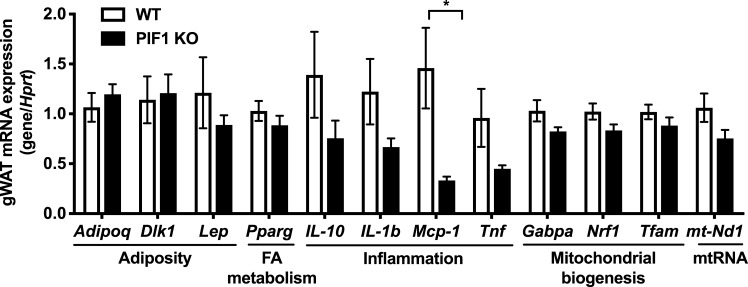
Transcript levels for inflammatory markers were decreased in adipose tissue from Western diet (WD)-fed PIF1 KO females. Gonadal white adipose tissue (gWAT) cDNA from WD-fed, WT and PIF1 KO mice was used to measure transcripts for genes involved in adiposity, fatty acid (FA) metabolism, inflammation, mitochondrial biogenesis and mitochondrial RNA. mRNA expression relative to the hypoxanthine-guanine phosphoribosyltransferase (*Hprt*) reference gene. All data are mean ± SEM; n = 8–12 mice per group; pvalues determined by Student’s T-test, *p<0.05.

As fat mass was increased in WD-fed PIF1 KO females compared with WD-fed WT mice, we assessed whether key markers of obesity pathogenesis were altered in gonadal white adipose tissue (cohort from Fig 1A). No differences in mRNA expression of selected adiposity, fatty acid metabolism, mitochondrial biogenesis or mitochondrial RNA genes (*Adipoq*, *Dlk1*, *Lep*, *Pparg*, *Gabpa*, *Nrf1*, *Tfam*, and *mt-Nd1*) were observed between groups (Fig 6). Interestingly, despite the increased adiposity in WD-fed PIF1 KO females, which is commonly associated with adipose tissue inflammation [26–30], mRNA expression of *Mcp-1* was four-fold downregulated in PIF1 KO mice, whereas other inflammatory genes (*Il-10*, *Il-1b*, *and Tnf*) trended towards lower expression in adipose tissue (Fig 6). To examine systemic inflammation, we measured serum concentrations of pro-inflammatory cytokines, but detected no differences between PIF1 KO and WT mice fed a long-term WD (Table 2). No differences in PIF1 transcript numbers in adipose from regular chow and WD-fed female WT mice were detected (S3 Fig).

**Table 2:** Cytokines measured in serum from Western diet-fed, WT and PIF1 KO female mice.

Cytokine	WT	PIF1 KO
IL-1α (ng/mL)	9.9 ± 2.0	8.0 ± 1.6
IL-1β (ng/mL)	206.2 ± 60.6	124.2 ± 23.4
IL-12 (ng/mL)	423.9 ± 98.2	695.2 ± 85.7
Eotaxin (ng/mL)	545.6 ± 143.6	524.1 ± 82.0
G-CSF (ng/mL)	31.5 ± 23.4	42.6 ± 10.2
IFN-γ (ng/mL)	20.6 ± 6.1	21.2 ± 3.4
MCP-1 (ng/mL)	166.4 ± 41.2	134.1 ± 34.5
MIP-1β (ng/mL)	29.7 ± 1.9	21.5 ± 3.6
TNF-α (ng/mL)	65.5 ± 18.6	52.3 ± 12.8
IL-12p70 (ng/mL)	49.4 ± 16.4	42.9 ± 9.1

All values are mean ± SEM; n = 3–12 per group.

## Discussion

While we have previously shown that PIF1 KO mice gain weight on a regular chow diet, it was not known whether PIF1 KO mice develop metabolic alterations under WD-induced weight gain. WD-fed PIF1 KO females had increased body weight and fat mass, but, paradoxically, similar fasting blood glucose concentrations and glucose tolerance compared with WD-fed WT females. Interestingly, the PIF1 KO females also had decreased expression of adipose tissue inflammatory markers. WD-fed PIF1 KO females developed a mild hepatic steatosis that appeared independent of weight gain, and without severe gene expression derangement.

The finding that WD-fed PIF1 KO females diverged in weight from WT females, while the males gained weight at a similar rate, was unexpected. The C57BL/6J strain used to generate the PIF1 KO mice has a sex-specific response to WD feeding [31, 32]. Specifically, female C57BL/6J mice gain less weight and are generally better protected against WD-induced metabolic syndrome than males [33]. Thus, our findings suggest that WD-fed PIF1 KO females are susceptible to diet-induced weight gain or lose protection against weight gain. Surprisingly, despite increased body weight and adiposity, there were no differences in fasting blood glucose or glucose tolerance in WD-fed PIF1 KO females compared with controls. The dissociation between obesity and metabolic dysfunction in WD-fed PIF1 KO female mice is reminiscent of metabolically healthy obesity [34, 35]. The decreased expression of some adipose tissue inflammatory markers in WD-fed PIF1 KO females compared with WD-fed WT controls may account for some aspects of the healthy-obese phenotype but requires further investigation. Similarly, the absence of changes in fasting blood glucose concentrations and glucose tolerance despite increased hepatic steatosis in WD-fed PIF1 KO females was surprising given the strong, positive association between liver fat, hyperglycemia and hepatic insulin resistance [36–38]. Future studies using isotopic infusion to measure rates of hepatic glucose production and insulin sensitivity directly, such as the hyperinsulinemic-euglycemic clamp, will be necessary to resolve this apparent paradox.

In addition to PIF1 helicase, experiments have been conducted in transgenic *Twinkle* mtDNA replication helicase mice [39]. TWINKLE is a nuclear-encoded, mtDNA replication helicase where specific mutations can cause autosomal dominant progressive external ophthalmoplegia (adPEO), a neurological disease associated with mtDNA deletions that cause progressive neuropathy with sensorineural loss [40]. Expression of a transgenic copy of an adPEO *Twinkle* allele in mice causes the accumulation of mtDNA deletions in the muscle and brain (mtDNA “Deletor” mice). Despite the presence of mtDNA deletions, Deletor mice do not display decreased physical performance or increased body weight [39]. These results from the Deletor mice suggest that the mtDNA deletions in the skeletal muscle of PIF1 KO mice previously described [4] do not contribute to the decreased activity and increased body weight shown in the current study.

The likely mechanism of increased weight gain in WD-fed PIF1 KO females was decreased locomotor activity. Both light and dark cycle activities were significantly reduced in PIF1 KO female mice after just four weeks of WD feeding when body weight differences were apparent, but modest. Additionally, there was a trend of decreased activity (25% reduction) after just two weeks of WD feeding prior to gross differences in body weight (S1C Fig). The importance of this two-week time point is that it was a weight-matched (pre-weight gain) cohort [41] that was evaluated pre- and post-WD exposure. Notably, when the activity trend is analyzed across all experiments and timepoints in the study, we find a statistically significant difference between WT and PIF1 during (S1F Fig). In the absence of pre-weight divergence hyperphagia (S1E Fig), the evidence best aligns with the conclusion that there is an interaction between PIF1 deficiency and Western diet that modulates locomotor activity to accelerate weight gain. By extension, we speculate that a PIF1 deficiency in humans might only cause obesity on Western diets.

The current study improves upon our prior understanding of weight gain in PIF1 KO mice [4], showing a dietary influence and sex-based differential response, but there are multiple outstanding questions remaining. We do not know the tissues or cell types that are crucial for regulating this interaction between PIF1 deficiency and Western diet, which could be limited to the central nervous system, liver, skeletal muscle, or thermal regulation (i.e. brown fat). We do not understand the reason for the sex-based response and potential role of estrogen signaling in this process. Furthermore, we do not know whether the key function of PIF1 in the phenotypes reported here are due to the nuclear or mitochondrial functions of PIF1. These questions highlight the need for new mouse models, such as tissue specific PIF1 knock-outs and nuclear and mitochondrial PIF1 knock-outs to further interrogate the role of this helicase in metabolism.

### Study limitations

There are potential limitations to any animal study, here we identify a few potential confounders of these results. Data are presented as intact single cohort studies within individual panels, with the exception of female mouse weight trends of WT vs PIF1 KO animals in S1F Fig and PIF1 transcript levels in S3A and S3B Fig, which are composites of two distinct studies. Comparison across panels, such as in Fig 4, were not part of the study design. Also, WT and PIF1 KO mice were bred at the University of Pittsburgh, but when WT mice numbers were limiting, WT mice were purchased from Jackson Laboratories. This action was justified within the study because heterozygous PIF1 KO mice were continuously backcrossed to WT C57BL/6J, which are replaced annually with mice from Jackson Laboratories, but yet unidentified effects on the cohort cannot be ruled out. Further, differential sensitivity to extent of animal handling, number of animals per cage, or other stress exposures has not been eliminated as a contributor to these phenotypes. As with most animal ablation studies, it should be acknowledged that mouse strain may be contributing to any differences observed.

## Supporting information

S1 Table List of primers used to measure gene expression in RNA isolated from white adipose tissue and liver. (DOCX)

S1 Fig Locomotor activity from short-term, Western diet (WD)-fed PIF1 KO mice was decreased prior to weight divergence between WD-fed WT and WD-fed PIF1 KO females. A) Locomotor activity, and B) whole-body energy expenditure were measured in 2-month-old WT and PIF1 KO females. The same mice were then placed on a short-term WD for 2 weeks to measure C) locomotor activity, D) whole-body energy expenditure, and E) daily food intake. F) Locomotor activity of the combined short- and long-term WD cohorts. All data are mean ± SEM; n = 8 mice per group; panel F analyzed by Two-way ANOVA detected genotype differences in WD samples, ***p<0.001, while Sidak’s multiple comparison test indicated 4-weeks to be significantly different, *p<0.05; kcal/h = kilocalories per hour; kg TBW = kilograms total body weight.
(EPS)

S2 Fig Western diet (WD) feeding did not affect mitochondrial DNA (mtDNA) copy content or mitochondrial complex I activity in regular chow- and WD-fed PIF1 KO mice. A) Total liver DNA from WD-fed, WT and PIF1 KO mice was used to measure mtDNA copy content. B) Liver homogenate supernatants from WD-fed, WT and PIF1 KO mice were used to assess mitochondrial complex I activity. All data are mean ± SEM; n = 8–12 mice per group.
(EPS)

S3 Fig PIF1 expression levels in liver and gonadal WAT adipose of regular chow- and western diet-fed WT mice. Liver and gonadal WAT cDNA was used to measure PIF1 transcript levels from A) regular chow-fed mice and B) Western diet-fed mice. Regular chow-fed mice were 7-months-old at sacking and WD-fed were 6 months-old. Data shown are mean ± SEM; n = 4–6 mice per group; Two-way Anova for regular chow and WD-fed mice detected interaction of gender and genotype only in WD samples, pvalue**<0.01, while Tukey’s post-hoc analysis detected difference of male WT liver from all other WD samples, ***p<0.001.
(EPS)

## References

[1] Ng M, Fleming T, Robinson M, Thomson B, Graetz N, Margono C, et al Global, regional, and national prevalence of overweight and obesity in children and adults during 1980–2013: a systematic analysis for the Global Burden of Disease Study 2013. Lancet. 2014;384(9945):766–81. Epub 2014/06/02. 10.1016/S0140-6736(14)60460-8 24880830 PMC4624264

[2] Llewellyn CH, Trzaskowski M, Plomin R, Wardle J. Finding the missing heritability in pediatric obesity: the contribution of genome-wide complex trait analysis. Int J Obes (Lond). 2013;37(11):1506–9. Epub 2013/03/27. 10.1038/ijo.2013.30 23528754 PMC3826033

[3] Twig G, Afek A, Derazne E, Tzur D, Cukierman-Yaffe T, Gerstein HC, et al Diabetes risk among overweight and obese metabolically healthy young adults. Diabetes Care. 2014;37(11):2989–95. Epub 2014/08/21. 10.2337/dc14-0869 . 25139886

[4] Bannwarth S, Berg-Alonso L, Aug?? Gl, Fragaki K, Kolesar JE, Lespinasse Fo, et al Inactivation of Pif1 helicase causes a mitochondrial myopathy in mice. Mitochondrion. 2016;30:126–37. 10.1016/j.mito.2016.02.005 . 26923168 PMC5454384

[5] Mateyak MK, Zakian VA. Human PIF helicase is cell cycle regulated and associates with telomerase. Cell cycle (Georgetown, Tex). 2006;5:2796–804. 10.4161/cc.5.23.3524 . 17172855

[6] Kazak L, Reyes A, Duncan AL, Rorbach J, Wood SR, Brea-Calvo G, et al Alternative translation initiation augments the human mitochondrial proteome. Nucleic Acids Research. 2013;41:2354–69. 10.1093/nar/gks1347 . 23275553 PMC3575844

[7] Schulz VP, Zakian VA. The saccharomyces PIF1 DNA helicase inhibits telomere elongation and de novo telomere formation. Cell. 1994;76:145–55. . 8287473 10.1016/0092-8674(94)90179-1

[8] Mangahas JL, Alexander MK, Sandell LL, Zakian VA. Repair of chromosome ends after telomere loss in Saccharomyces. Mol Biol Cell. 2001;12(12):4078–89. Epub 2001/12/12. 10.1091/mbc.12.12.4078 11739802 PMC60777

[9] Boule JB, Zakian VA. Roles of Pif1-like helicases in the maintenance of genomic stability. Nucleic Acids Res. 2006;34(15):4147–53. Epub 2006/08/29. 10.1093/nar/gkl561 16935874 PMC1616966

[10] Vega LR, Phillips JA, Thornton BR, Benanti JA, Onigbanjo MT, Toczyski DP, et al Sensitivity of yeast strains with long G-tails to levels of telomere-bound telomerase. PLoS Genetics. 2007;3:e105 10.1371/journal.pgen.0030105 . 17590086 PMC1892048

[11] Paeschke K, Bochman ML, Garcia PD, Cejka P, Friedman KL, Kowalczykowski SC, et al Pif1 family helicases suppress genome instability at G-quadruplex motifs. Nature. 2013;497:458–62. 10.1038/nature12149 . 23657261 PMC3680789

[12] Schuldt A. DNA replication: Pif1 overcomes a quadruplex hurdle. Nature Reviews Molecular Cell Biology. 2011;12:402–3. 10.1038/nrm3142 . 21673725

[13] Snow BE, Mateyak M, Paderova J, Wakeham A, Iorio C, Zakian V, et al Murine Pif1 interacts with telomerase and is dispensable for telomere function in vivo. Molecular and Cellular Biology. 2007;27:1017–26. 10.1128/MCB.01866-06 . 17130244 PMC1800700

[14] Kolesar JE, Wang CY, Taguchi YV, Chou S-H, Kaufman BA. Two-dimensional intact mitochondrial DNA agarose electrophoresis reveals the structural complexity of the mammalian mitochondrial genome. Nucleic Acids Research. 2013;41:e58—e. 10.1093/nar/gks1324 . 23275548 PMC3575812

[15] Livak KJ, Schmittgen TD. Analysis of relative gene expression data using real-time quantitative PCR and. Methods. 2001;25:402–8. 10.1006/meth.2001.1262 . 11846609

[16] Shiva S, Sack MN, Greer JJ, Duranski M, Ringwood LA, Burwell L, et al Nitrite augments tolerance to ischemia/reperfusion injury via the modulation of mitochondrial electron transfer. J Exp Med. 2007;204(9):2089–102. Epub 2007/08/08. 10.1084/jem.20070198 17682069 PMC2118713

[17] Kolesar JE, Safdar A, Abadi A, MacNeil LG, Crane JD, Tarnopolsky MA, et al Defects in mitochondrial DNA replication and oxidative damage in muscle of mtDNA mutator mice. Free Radical Biology and Medicine. 2014;75:241–51. 10.1016/j.freeradbiomed.2014.07.038 . 25106705

[18] Assessment of feeding behavior in laboratory mice, (2010). 10.1016/j.cmet.2010.06.001 PMC2916675 20620991

[19] Rui L. Energy metabolism in the liver. Compr Physiol. 2014;4(1):177–97. Epub 2014/04/03. 10.1002/cphy.c130024 24692138 PMC4050641

[20] Gavrilova O, Haluzik M, Matsusue K, Cutson JJ, Johnson L, Dietz KR, et al Liver peroxisome proliferator-activated receptor gamma contributes to hepatic steatosis, triglyceride clearance, and regulation of body fat mass. J Biol Chem. 2003;278(36):34268–76. Epub 2003/06/14. 10.1074/jbc.M300043200 . 12805374

[21] Inoue M, Ohtake T, Motomura W, Takahashi N, Hosoki Y, Miyoshi S, et al Increased expression of PPARgamma in high fat diet-induced liver steatosis in mice. Biochem Biophys Res Commun. 2005;336(1):215–22. Epub 2005/08/30. 10.1016/j.bbrc.2005.08.070 . 16125673

[22] Matsusue K, Haluzik M, Lambert G, Yim SH, Gavrilova O, Ward JM, et al Liver-specific disruption of PPARgamma in leptin-deficient mice improves fatty liver but aggravates diabetic phenotypes. J Clin Invest. 2003;111(5):737–47. Epub 2003/03/06. 10.1172/JCI17223 12618528 PMC151902

[23] Matsusue K, Kusakabe T, Noguchi T, Takiguchi S, Suzuki T, Yamano S, et al Hepatic steatosis in leptin-deficient mice is promoted by the PPARgamma target gene Fsp27. Cell Metab. 2008;7(4):302–11. Epub 2008/04/09. 10.1016/j.cmet.2008.03.003 18396136 PMC2587176

[24] Moran-Salvador E, Lopez-Parra M, Garcia-Alonso V, Titos E, Martinez-Clemente M, Gonzalez-Periz A, et al Role for PPARgamma in obesity-induced hepatic steatosis as determined by hepatocyte- and macrophage-specific conditional knockouts. FASEB J. 2011;25(8):2538–50. Epub 2011/04/22. 10.1096/fj.10-173716 . 21507897

[25] Xu Z, Chen L, Leung L, Yen TS, Lee C, Chan JY. Liver-specific inactivation of the Nrf1 gene in adult mouse leads to nonalcoholic steatohepatitis and hepatic neoplasia. Proc Natl Acad Sci U S A. 2005;102(11):4120–5. Epub 2005/03/02. 10.1073/pnas.0500660102 15738389 PMC554825

[26] Kamei N, Tobe K, Suzuki R, Ohsugi M, Watanabe T, Kubota N, et al Overexpression of monocyte chemoattractant protein-1 in adipose tissues causes macrophage recruitment and insulin resistance. J Biol Chem. 2006;281(36):26602–14. Epub 2006/07/01. 10.1074/jbc.M601284200 . 16809344

[27] Kanda H, Tateya S, Tamori Y, Kotani K, Hiasa K, Kitazawa R, et al MCP-1 contributes to macrophage infiltration into adipose tissue, insulin resistance, and hepatic steatosis in obesity. J Clin Invest. 2006;116(6):1494–505. Epub 2006/05/13. 10.1172/JCI26498 16691291 PMC1459069

[28] Rosen ED, Spiegelman BM. Adipocytes as regulators of energy balance and glucose homeostasis. Nature. 2006;444(7121):847–53. Epub 2006/12/15. 10.1038/nature05483 17167472 PMC3212857

[29] Sartipy P, Loskutoff DJ. Monocyte chemoattractant protein 1 in obesity and insulin resistance. Proc Natl Acad Sci U S A. 2003;100(12):7265–70. Epub 2003/05/21. 10.1073/pnas.1133870100 12756299 PMC165864

[30] van der Heijden RA, Sheedfar F, Morrison MC, Hommelberg PP, Kor D, Kloosterhuis NJ, et al High-fat diet induced obesity primes inflammation in adipose tissue prior to liver in C57BL/6j mice. Aging (Albany NY). 2015;7(4):256–68. Epub 2015/05/17. 10.18632/aging.100738 25979814 PMC4429090

[31] Gallou-Kabani C, Vige A, Gross MS, Rabes JP, Boileau C, Larue-Achagiotis C, et al C57BL/6J and A/J mice fed a high-fat diet delineate components of metabolic syndrome. Obesity (Silver Spring). 2007;15(8):1996–2005. Epub 2007/08/23. 10.1038/oby.2007.238 . 17712117

[32] Hwang LL, Wang CH, Li TL, Chang SD, Lin LC, Chen CP, et al Sex differences in high-fat diet-induced obesity, metabolic alterations and learning, and synaptic plasticity deficits in mice. Obesity (Silver Spring). 2010;18(3):463–9. Epub 2009/09/05. 10.1038/oby.2009.273 . 19730425

[33] Pettersson US, Walden TB, Carlsson PO, Jansson L, Phillipson M. Female mice are protected against high-fat diet induced metabolic syndrome and increase the regulatory T cell population in adipose tissue. PLoS One. 2012;7(9):e46057 Epub 2012/10/11. 10.1371/journal.pone.0046057 23049932 PMC3458106

[34] Stefan N, Haring HU, Hu FB, Schulze MB. Metabolically healthy obesity: epidemiology, mechanisms, and clinical implications. Lancet Diabetes Endocrinol. 2013;1(2):152–62. Epub 2014/03/14. 10.1016/S2213-8587(13)70062-7 . 24622321

[35] Phillips CM. Metabolically healthy obesity: definitions, determinants and clinical implications. Rev Endocr Metab Disord. 2013;14(3):219–27. Epub 2013/08/10. 10.1007/s11154-013-9252-x . 23928851

[36] Samuel VT, Shulman GI. Mechanisms for insulin resistance: common threads and missing links. Cell. 2012;148(5):852–71. Epub 2012/03/06. 10.1016/j.cell.2012.02.017 22385956 PMC3294420

[37] Samuel VT, Shulman GI. The pathogenesis of insulin resistance: integrating signaling pathways and substrate flux. J Clin Invest. 2016;126(1):12–22. Epub 2016/01/05. 10.1172/JCI77812 26727229 PMC4701542

[38] Samuel VT, Liu ZX, Qu X, Elder BD, Bilz S, Befroy D, et al Mechanism of hepatic insulin resistance in non-alcoholic fatty liver disease. J Biol Chem. 2004;279(31):32345–53. Epub 2004/05/29. 10.1074/jbc.M313478200 . 15166226

[39] Tyynismaa H, Mjosund KP, Wanrooij S, Lappalainen I, Ylikallio E, Jalanko A, et al Mutant mitochondrial helicase Twinkle causes multiple mtDNA deletions and a late-onset mitochondrial disease in mice. Proceedings of the National Academy of Sciences of the United States of America. 2005;102:17687–92. 10.1073/pnas.0505551102 . 16301523 PMC1308896

[40] Fratter C, Gorman GS, Stewart JD, Buddles M, Smith C, Evans J, et al The clinical, histochemical, and molecular spectrum of PEO1 (Twinkle)-linked adPEO. Neurology. 2010;74(20):1619–26. Epub 2010/05/19. 10.1212/WNL.0b013e3181df099f 20479361 PMC2875130

[41] Kaiyala KJ, Schwartz MW. Toward a more complete (and less controversial) understanding of energy expenditure and its role in obesity pathogenesis. Diabetes. 2011;60(1):17–23. Epub 2011/01/05. 10.2337/db10-0909 21193735 PMC3012169

