# Supplementary material for: Petite Integration Factor 1 (PIF1) helicase deficiency increases weight gain in Western diet-fed female mice without increased inflammatory markers or decreased glucose clearance
Source: PLoS One. 2019 May 28;14(5):e0203101. doi: 10.1371/journal.pone.0203101 (PMC6538152; doi:10.1371/journal.pone.0203101)
Supplement: S1 Table — (DOCX) [file pone.0203101.s001.docx]

**Table S1. List of primers used to measure gene expression in RNA isolated from white adipose tissue and liver.**

| **Primer name** | **Sequence** |
| --- | --- |
| Acc1 FWD  Acc1 REV | \| GCGGGAGGAGTTCCTAATTC \| \| --- \| \| GTGGATTTTCTTCTTGACCAGG \| |
| Acc2 FWD  Acc2 REV | \| ACTGGACCTGCACAGAGAT \| \| --- \| \| CCAAACCGAGTGACAAAC \| |
| Act B FWD  Act B REV | GATTACTGCTCTGGCTCCTAG  GACTCATCGTACTCCTGCTTG |
| Adipoq FWD  Adipoq REV | TGGCCACTTTCTCCTCATTTC  CATGACTGGGCAGGATTAAGAG |
| Cd36 FWD  Cd36 REV | \| TGGAGCTGTTATTGGTGCAG \| \| --- \| \| TGGGTTTTGCACATCAAAGA \| |
| Cpt1a FWD  Cpt1a REV | CTCTGCTGCATGGTAGATGTT  GCTCTGCGTTTATGCCTATCT |
| Dlk1 FWD  Dlk1 REV | GATTCTGCGAGGCTGACAA  TGCAGACTCCATTGACACAG |
| Fabp2 FWD  Fabp2 REV | \| GTGGAAAGTAGACCGGAACGA \| \| --- \| \| CCATCCTGTGTGATTGTCAGTT \| |
| Fatp5 FWD  Fatp5 REV | \| GGTTTTTGCATTCCTGTGGA \| \| --- \| \| GAAGGGTTGGTTCTTTCGAA \| |
| Gabpa FWD  Gabpa REV | GCCAGCCAAGAGCAACAGATGAAT  GGACCGTTGCACTTTAGCTGCTTT |
| G6pc FWD  G6pc REV | \| CAGTGGTCGGAGACTGGTTC \| \| --- \| \| TATAGGCACGGAGCTGTTGC \| |
| Hprt FWD  Hprt REV | GGCCAGACTTTGTTGGATTTG  TGCGCTCATCTTAGGCTTTGT |
| IL-1b FWD  Il-1b REV | \| GGTCAAAGGTTTGGAAGCAG \| \| --- \| \| TGTGAAATGCCACCTTTTGA \| |
| Il-6 FWD  Il-6 REV | \| ACCAGAGGAAATTTTCAATAGGC \| \| --- \| \| TGATGCACTTGCAGAAAACA \| |
| Il-10 FWD  Il-10 REV | \| TGTCAAATTCATTCATGGCCT \| \| --- \| \| ATCGATTTCTCCCCTGTGAA \| |
| Ldlr FWD  Ldlr REV | \| CGCGGATCTGATGCGTCGCT \| \| --- \| \| CGGCCCTGGCAGTTCTGTGG \| |
| Lep FWD  Lep REV | \| GGACGGTAACGGGAATGTATG \| \| --- \| \| ACGTTGTCTAGGGGGTACTTAAA \| |
| Lxra FWD  Lxra REV | \| CTCAATGCCTGATGTTTCTCCT \| \| --- \| \| TCCAACCCTATCCCTAAAGCAA \| |
| Mcp1 FWD  Mcp1 REV | \| TTGGGATCATCTTGCTGGT \| \| --- \| \| CCTGCTGTTCACAGTTGCC \| |
| Nd1 FWD  Nd1 REV | CCATTTGCAGACGCCATAAA  GAGTGATAGGGTAGGTGCAATAA |
| Nrf1 FWD  Nrf1 REV | ACAGATAGTCCTGTCTGGGGAAA  TGGTACATGCTCACAGGGATCT |
| Pck1 FWD  Pck1 REV | \| GTGCTGGAGTGGATGTTCGG \| \| --- \| \| CTGGCTGATTCTCTGTTTCAGG \| |
| Ppara FWD  Ppara REV | AAGAACCTGAGGAAGCCGTTCTGT  GCAGCCACAAACAGGGAAATGTCA |
| Pparg FWD  Pparg REV | ACATAAAGTCCTTCCCGCTGACCA  AAATTCGGATGGCCACCTCTTTGC |
| Ppargc1a FWD  Ppargc1a REV | ACTATGAATCAAGCCACTACAGAC  TTCATCCCTCTTGAGCCTTTCG |
| Scd1 FWD  Scd1 REV | \| GGAACTAGTGAGGTTGAGGGG \| \| --- \| \| TGGCCACAAAGCAAAGAAG \| |
| Srebp FWD  Srebp REV | \| GAACAGACACTGGCCGAGAT \| \| --- \| \| GAGGCCAGAGAAGCAGAAGAG \| |
| Tfam FWD  Tfam REV | AGCTTCCAGGAGGCAAGGATGAT  ACTTCAGCCATCTGCTCTCAA |
| Tnf FWD  Tnf REV | \| AGGGTCTGGGCCATAGAACT \| \| --- \| \| CCACCACGCTCTTCTGTCTAC \| |
